# A Potent Trivalent Sialic Acid Inhibitor of Adenovirus Type 37 Infection of Human Corneal Cells**

**DOI:** 10.1002/anie.201101559

**Published:** 2011-06-06

**Authors:** Sara Spjut, Weixing Qian, Johannes Bauer, Rickard Storm, Lars Frängsmyr, Thilo Stehle, Niklas Arnberg, Mikael Elofsson

**Affiliations:** Department of Chemistry, Umeå Centre for Microbial Research (UCMR) and Laboratory for Molecular Infection Medicine Sweden (MIMS)Umeå University, 90187 Umeå (Sweden); Interfaculty Institute of Biochemistry, University of Tübingen72076 Tübingen (Germany); Department of Clinical Microbiology, Division of Virology, MIMS, Umeå University90187 Umeå (Sweden); Department of Pediatrics, Vanderbilt University School of MedicineNashville 37232, TN (USA)

**Keywords:** adenoviruses, antiviral agents, crystal-structure elucidation, sialic acids, surface plasmon resonance

Viruses of the *Adenoviridae* family are widespread in society and are associated with a wide variety of clinical symptoms in humans, including respiratory, gastrointestinal, and ocular diseases.[1] Epidemic keratoconjunctivitis (EKC) is a severe ocular infection and is caused by the highly contagious adenoviruses Ad8, Ad19, and Ad37.[1] Besides keratitis and conjunctivitis, other common symptoms of EKC are pain, lacrimation, red and swollen eyes, as well as decreased vision that may last for months or even years.[1] No antiviral drugs are currently available for the treatment of EKC or any other infection caused by adenoviruses. The initial event leading to EKC is binding of the viruses to glycans that contain sialic acid moieties on epithelial cells in the cornea or conjunctiva through trimeric fiber structures extending from the viral particles.[2, 3] The receptor-binding domain, the fiber knob, is located at the C terminus of each fiber and contains three separate pockets that each can accommodate one sialic acid residue. Ad37 was recently shown to bind to cell-surface glycoproteins carrying a glycan structure similar to the GD1a ganglioside.[4] The GD1a glycan is a branched hexasaccharide with a terminal sialic acid residue on each of its two arms. Structural studies showed that the two sialic acid moieties dock into two of three sialic acid binding sites in the trimeric knob of the Ad37 fiber protein. Most likely, multiple fiber proteins simultaneously engage several host-cell epitopes containing terminal sialic acids; internalization and subsequent infection follow. If these sialic acid–protein interactions can be blocked, for example, by a multivalent sialic acid conjugate, infection might be prevented.

To date, several carbohydrate-based or glycomimetic drugs have reached the market; however, the development of additional therapies is still hampered by challenges such as poor absorption and/or rapid elimination.[5] The topical administration of sialic acid conjugates directly to the eye, that is, the site of infection, circumvents many of the pharmacokinetic hurdles and has the potential to prevent or even cure EKC. In the search for new antiviral substances against Ad37, we synthesized and evaluated multivalent human serum albumin (HSA) conjugates of both 3′-sialyllactose and sialic acid as adenoviral inhibitors.[6, 7] These conjugates efficiently inhibited Ad37 cell attachment and the subsequent infection of human corneal epithelial (HCE) cells. Both types of conjugates were equally efficient as Ad37 inhibitors.[6, 7] From the crystal structure of the fiber-knob protein as a complex with sialyllactose, it was evident that the sialic acid acetamide group is positioned in a relatively large hydrophobic pocket.[8] To improve the potency of the more advantageous multivalent sialic acid conjugates, we used structure-based design and synthesized a library of ten *N*-acyl-modified sialic acid derivatives with the overall goal of improving hydrophobic interactions and thus affinity and efficacy.[9] Unfortunately, none of the designed conjugates were as potent as the original sialic acid–HSA conjugate, although X-ray crystallography revealed that the modified saccharides interacted with the fiber-knob protein as expected.[9]

**Scheme 1:**
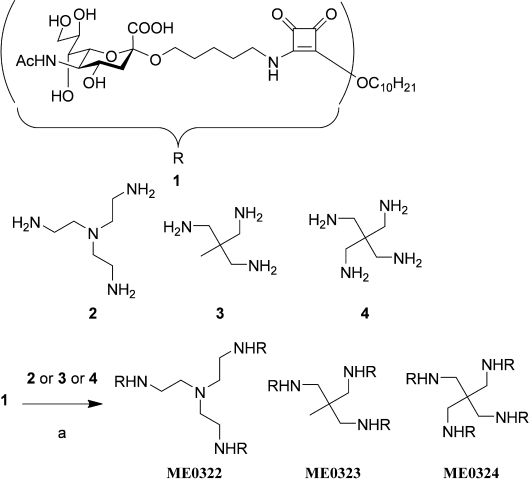
Synthesis of tri- and tetravalent sialic acid compounds. The sialic acid squaric decyl ester **1** was coupled to the scaffolds **2, 3**, and **4** to form the compounds **ME0322, ME0323** and **ME0324**: a) *N,N*-diisopropylethylamine, MeOH, room temperature, 2–8 days, 30–69 %.

On the basis of the structural features of the interaction of the GD1a glycan with the Ad37 knob,[4] and with our previous results[6, 7, 9] in mind, we then designed and synthesized sialic acid containing compounds by using small non-protein scaffolds. The crystal structure of the fiber-knob protein shows that the three known sialic acid binding sites are separated by distances of about 10 Å. We therefore considered the design of a compact and rigid scaffold decorated with three correctly positioned sialic acids as too complex. Instead we selected the three small and flexible scaffolds tris(2-aminoethyl)amine (**2**), 2-(aminomethyl)-2-methyl-1,3-propanediamine (**3**), and 2,2-diaminomethyl-1,3-propanediamine (**4**) for conjugation with the sialic acid derivative **1**[7] through the use of squaric acid chemistry and thus prepared **ME0322, ME0323**, and **ME0324** in modest to good yields (Scheme 1; see also the Supporting Information). We reasoned that longer flexible spacers would enable all three binding pockets to be occupied simultaneously by one molecule.

**Figure 1:**
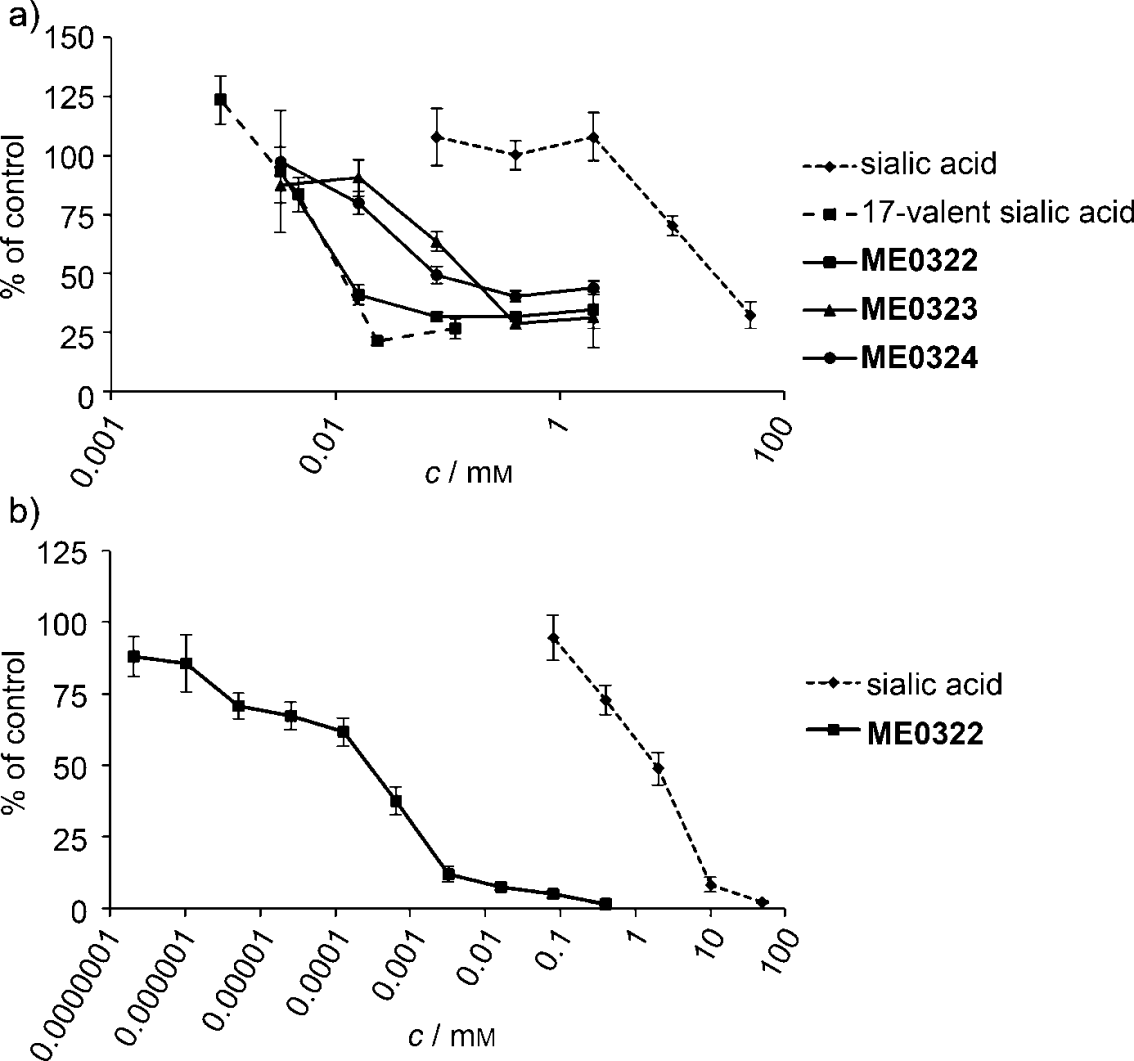
Effect of sialic acid containing compounds on adenovirus type 37 binding to and infection of human corneal cells. a) Extent of virion binding in the presence of inhibitors at different concentrations. b) Extent of infection at different concentrations of the inhibitors sialic acid and **ME0322**. The control is the value obtained for binding or infection in the absence of an inhibitor.

The effects of **ME0322, ME323, ME0324**, sialic acid, and a 17-valent sialic acid–HSA conjugate were first evaluated in virus-binding experiments (see the Supporting Information).[6, 7, 9] **ME0322, ME0323**, and **ME0324** all inhibited the attachment of Ad37 virions to HCE cells in a dose-dependent manner and were at least two orders of magnitude more effective than sialic acid. Importantly, the most potent compound, **ME0322**, was as efficient as the 17-valent sialic acid–HSA conjugate (Figure 1a).[7] To firmly establish the potential of **ME0322** as an anti-adenoviral agent, we evaluated the compound further in a cell-based infection assay (see the Supporting Information).[6, 7, 9] Compound **ME0322** proved to be very potent and inhibited the infection of HCE cells by Ad37 virions with an IC_50_ value of 0.38 μm (Figure 1b). Remarkably, the trivalent compound **ME0322** was approximately four orders of magnitude more potent than sialic acid (Figure 1b) and substantially more potent than 3′-sialyllactose–HSA and sialic acid–HSA conjugates.[6, 9]

**Figure 2:**
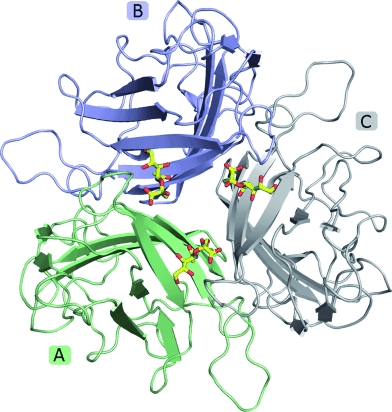
Overall structure of the Ad37 fiber head as a complex with **ME0322**. The three Ad37 chains A, B, and C are shown as ribbon tracings and colored green, blue, and gray, respectively. The terminal sialic acid moietes of **ME0322** are bound on top of the fiber head and are shown in stick representation, with carbon atoms in yellow, oxygen atoms in red, and nitrogen atoms in blue. The spacers and scaffold are not shown.

To determine the structural features of the Ad37 fiber knob–**ME0322** interaction, we solved the crystal structure of this complex at a resolution of 2.4 Å (Figure 2; see also the Supporting Information, including Table S1). Only the terminal sialic acid residues of **ME0322** are visible in the final electron-density map (see Figure S1 in the Supporting Information). We did not observe electron density for the rest of the compound, probably because the flexible linkers do not make defined contacts with the protein. It is, however, likely that all three sialic acids in a fiber knob belong to the same trivalent compound. Analysis of the crystal packing shows that the distances between sialic acid binding sites in different knobs are too large to be bridged by a single **ME0322** molecule (data not shown). The binding of all sialic acid residues and their interactions with their respective protein chains are identical to the previously established binding mode between the sialic acid residues of sialyl-α(2,3)-lactose or the GD1a hexasaccharide and the Ad37 fiber knob.[4, 8]

Finally, we investigated the binding affinity of the trivalent sialic acid conjugate **ME0322** for Ad37 fiber knobs immobilized on a CM5 sensor chip by surface plasmon resonance (see the Supporting Information, including Figure S2). The binding was shown to fit to a simple one-to-one binding model, and the calculated *K*_d_ value for the interaction of the Ad37 fiber-knob protein with **ME0322** was calculated to be 14 μm. The interaction of the GD1a hexasaccharide with the Ad37 fiber-knob protein, on the other hand, follows a two-to-one binding model with *K*_d_=19 and 265 μm.[4] Our results suggest that the high-affinity interaction results from the occupation of two of the three sialic acid binding pockets by the two terminal sialic acids of one GD1a hexasaccharide, and that another, unknown site is engaged in the low-affinity interaction. **ME0322** and the GD1a hexasaccharide thus bind to the fiber-knob protein with similar affinities. Interestingly, the GD1a hexasaccharide is a poor inhibitor of cell infection (IC_50_=0.7 mm),[4] in contrast to **ME0322**, which is approximately four orders of magnitude more potent. The number of components and processes in the cell-based assay, however, makes it difficult to directly relate calculated affinities obtained by surface plasmon resonance analysis to potency in the infection assay.

In conclusion, we have synthesized tri- and tetravalent sialic acid compounds and evaluated them in an Ad37 cell-binding assay. The most promising trivalent compound, **ME0322**, was subsequently shown to be a very potent inhibitor of Ad37 infection of human ocular cells. Our functional and structural data show that **ME0322** efficiently blocks the adenovirus cell-binding protein, the fiber knob. Therefore, such compounds offer promise as antiviral drugs for the topical treatment of EKC.
