# Supplementary material for: A Potent Trivalent Sialic Acid Inhibitor of Adenovirus Type 37 Infection of Human Corneal Cells
Source: Angew Chem Int Ed Engl. 2011 Jun 6;50(29):6519–21. doi: 10.1002/anie.201101559 (PMC3210828; doi:10.1002/anie.201101559)
Supplement: Supplementary file 1 [file anie0050-6519-SD1.pdf]

Angewandte Chemie
Eine Zeitschrift der Gesellschaft Deutscher Chemiker

Supporting Information
© Wiley-VCH 2011
69451 Weinheim, Germany

A Potent Trivalent Sialic Acid Inhibitor of Adenovirus Type 37 Infection of Human Corneal Cells**

*Sara Spjut, Weixing Qian, Johannes Bauer, Rickard Storm, Lars Frängsmyr, Thilo Stehle, Niklas Arnberg, and Mikael Elofsson**

anie_201101559_sm_miscellaneous_information.pdf

SUPPORTING INFORMATION

Table of Contents

1. METHODS 2

General chemical methods and materials 2

Binding assay 4

Infection assay.. 4

Protein production and structure determination 5

Surface Plasmon Resonance 6

2. TABLE S1 7

3. FIGURE S1 AND S2 8

4. REFERENCES 10

1. METHODS

General chemical methods and materials

^1H and ^{13}C NMR spectra were recorded with a Bruker DRX-400 spectrometer at 400 MHz and 100 MHz, respectively at 298 K. Chemical shifts are referenced to solutions in D_2O [residual HOD (δ_H 4.79 ppm) as internal standard]. Chemical shifts and proton and carbon resonance assignments were obtained from COSY and ^1H-^{13}C-HMQC experiments. Proton resonances that could not be assigned and aromatic resonances are generally not reported. LCMS was carried out with a Waters LC system equipped with an Xterra C-18 column (50 × 19 mm, 5 µm, 125 Å), eluted with a linear gradient of CH_3CN in water, both of which contained formic acid (0.2 %). A flow rate of 1.5 ml/min was used and detection was performed at 214 and 254 nm, and with positive and negative electrospray mass analysis. Mass spectra were obtained on a Water micromass ZQ 2000 using positive and negative electrospray ionization. Preparative HPLC separations were performed on a Beckman System Gold HPLC, using a Kromasil C-18 column (250 × 20 mm, 5 µm, 100 Å) with a flow rate of 11 ml/min, detection at 214 nm and eluent system: A. aq. 0.1 % CF_3CO_2H, and B. 0.1 % CF_3CO_2H in CH_3CN. Analytical HPLC was performed on a Beckman System Gold HPLC, using a Kromasil C-18 column (250 × 24.6 mm, 5 µm, 100 Å) with a flow rate of 1.5 mL/min, detection at 214 nm, and eluent system: A. aq. 0.1 % CF_3CO_2H, and B. 0.1% CF_3CO_2H in CH_3CN. Thin layer chromatography (TLC) was carried out on Silica Gel F_{254} (Merck), with detection under UV light and development with aqueous sulfuric acid (10 %). Solutions were concentrated using rotary evaporation. MeOH was dried over 3Å molecular sieves. All other chemicals were used as received. Optical rotations were measured with a Perkin-Elmer 343 polarimeter at 20 °C.

Tris-{*N*-[4-((5-acetamido-3,5-dideoxy-D-glycero-a-D-galacto-2-nonylopyranosylonic acid)pentylamino)-2,3-dioxocyclobut-1-enyl]aminoethyl}amine (ME0322)

Tris(2-aminoethyl)amine (2.4 mg, 0.016 mmol) and **2** (62.0 mg, 0.098 mmol) were dissolved in methanol (1.6 ml) and *N,N*-diisopropylethylamine (0.050 ml, 0.29 mmol) was added. The mixture was stirred at room temperature for 2 days. Solvent was removed under vacuum and the residue was purified by preparative HPLC (condition: 0 % to 50 % CH_3CN in 60 min.) to give 17.7 mg (69 %) of **ME0322**. ^1H NMR (D_2O): δ 1.31-1.46 (m, 6H, 3×-$OCH_2CH_2CH_2NH$-), 1.51-1.66 (m, 12H, 3×-

$OCH_2CH_2CH_2CH_2CH_2NH$-), 1.71 (t, 3H, J_{H4eq} = 12.0 Hz, 3×$H3_{ax}$), 2.02 (s, 12H, 3×Ac), 2.68 (dd, 3H, J_{H4} = 4.0Hz, J_{H3ax} = 12.0 Hz, 3×$H3_{eq}$), 3.44-3.50 (m, 3H), 3.51-3.60 (m, 9H), 3.61-3.70 (m, 9H), 3.71-3.79 (m, 9H), 3.80-3.88 (m, 9H), 4.08 (s, 6H); ^{13}C NMR ($CDCl_3$): δ 22.0, 22.0, 28.3, 29.6, 38.7, 39.6, 44.3, 51.8, 54.3, 62.8, 64.4, 67.6, 68.2, 71.2, 72.8, 99.4, 167.4, 172.0, 172.1, 175.0, 181.2, 182.0; MS(ES+) calculated for $C_{66}H_{103}N_{10}O_{33}$($M$+$H^+$) 1563.67, found 1564.52; $[\alpha]_D^{20}$ -8.0° (c 1.0, H_2O).

Tris-{*N*-[4-((5-acetamido-3,5-dideoxy-D-glycero-a-D-galacto-2-nonylopyranosylonic acid) pentylamino)-2,3-dioxocyclobut-1-enyl]aminomethyl}ethane (ME0323)

2-Aminomethyl-2-methyl-1,3-propanediamine (4.36 mg, 0.019 mmol) and **3** (72.9 mg, 0.116 mmol) were dissolved in methanol (2.0 ml) and *N,N*-diisopropylethylamine (0.050 ml, 0.29 mmol) was added. The mixture was stirred at room temperature for 8 days. Solvent was removed under vacuum and the residue was purified by preparative HPLC (condition: 0 % to 50 % CH_3CN in 60 min.) to give **ME0323** in 15.4 mg (52 %). ^{1}H NMR (D_2O) δ 1.04 (s, 3H, -CH_3), 1.31-1.45 (m, 6H, 3×-$OCH_2CH_2CH_2NH$-), 1.50-1.66 (m, 12H, 3×-$OCH_2CH_2CH_2CH_2CH_2NH$-), 1.71 (t, 3H, J_{H4eq} = 12.0 Hz, 3×$H3_{ax}$), 2.00 (s, 12H, 3×Ac), 2.65 (dd, 3H, J_{H4} = 4.0 Hz, J_{H3ax} = 12.0 Hz, 3×$H3_{eq}$), 3.43-3.67 (m, 21H), 3.68-3.78 (m, 9H), 3.78-3.86 (m, 9H); ^{13}C NMR ($CDCl_3$): δ 22.0, 22.0, 28.3, 38.8, 39.7, 44.2, 51.8, 52.0, 62.8, 63.1, 64.5, 66.7, 67.7, 68.2, 70.1, 70.3, 71.2, 72.7, 95.4, 99.6, 168.2, 172.2, 173.6, 174.8, 175.0; MS(ES+) calculated for $C_{65}H_{100}N_9O_{33}$(M+H^+) 1534.63, found 1535.62; $[\alpha]_D^{20}$ –14.9° (c 0.5, H_2O).

Tetra-{*N*-[4-((5-acetamido-3,5-dideoxy-D-glycero-a-D-galacto-2-nonylopyranosylonic acid) pentylamino)-2,3-dioxocyclobut-1-enyl]aminomethyl}methane (ME0324)

Pentaerythrityl tetramine tetrahydrochloride (4.2 mg, 0.015 mmol) and **4** (75.6 mg, 0.12 mmol) were dissolved in methanol (2.0 ml) and *N,N*-diisopropylethylamine (0.070 ml, 0.40 mmol) was added. The mixture was stirred at room temperature for 8 days. Solvent was removed under vacuum and the residue was purified by preparative HPLC (condition: 5 % to 20 % CH_3CN in 90 min.) to give **ME0324** in 9.0 mg (30 %). ^{1}H NMR (D_2O): δ 1.32-1.47 (m, 8H, 4×-$OCH_2CH_2CH_2NH$-), 1.53-1.67 (m, 16H, 4×-$OCH_2CH_2CH_2CH_2CH_2NH$-), 1.71 (t, 4H, J_{H4eq} = 12.0 Hz, 4×$H3_{ax}$), 2.01 (s, 12H,

4×Ac), 2.67 (dd, 4H, J_{H4} = 4.0 Hz, J_{H3ax} = 12.0 Hz, 4×$H3_{eq}$), 3.44-3.65 (m, 20H), 3.67-3.87 (m, 32H); ^{13}C NMR ($CDCl_3$): δ 21.9, 22.0, 28.3, 29.7, 30.9, 38.8, 39.6, 44.2, 51.8, 52.0, 61.5, 62.8, 63.1, 64.5, 66.7, 67.6, 68.1, 68.2, 70.1, 70.4, 71.1, 72.8, 95.3, 99.4, 167.9, 171.9, 173.3, 175.0, 181.0, 182.0; MS(ES+) calculated for $C_{85}H_{129}N_{12}O_{44}$($M$+$H^+$) 2021.82, found 2023.63; $[\alpha]_D^{20}$ -6.1° (c 0.9, H_2O).

Binding assay

The assay was carried out essentially as described previously.[1-3] ^{35}S-labeled Ad37 virions (1×10^9 per well) were incubated in 50 µl binding buffer (BB: DMEM containing 1 % bovine serum albumin [Roche AB, Stockholm, Sweden] and HEPES [EuroClone, Milan, Italy], pH 7.5) at 4 °C, together with different concentrations of commercial sialic acid, tri- or tetravalent glycoconjugates **ME0322**, **ME0323**, and **ME0324** or 17-valent sialic acid-HSA in 96-well plates for one hour. The mixtures were transferred to V-shaped 96-well plates with 50 µl HCE cells in BB (2×10^5 cells per well) and incubated for one hour at 4 °C. After two washes in BB, the cell-associated radioactivity was measured in a Wallac 1409 liquid scintillation counter (Perkin Elmer). Data are presented as % of control, which is the value obtained in absence of inhibitor. Mean values and standard deviations (calculated with the Gauss approximation formula) are from duplicates, and experiments were reproduced three times, except the experiment with the 17-valent sialic acid-HSA conjugate that was only performed once.

Infection assay

The assay was carried out essentially as described previously.[1-3] Non-labeled Ad37 virions (0.5×10^9 per well in 300 µl BB) were incubated at 4 °C in 24-well plates together with different concentrations of **ME0322** or commercial sialic acid. After one hour the mixtures were transferred to HCE cells grown as monolayers in 24-well plates (2×10^5 cells per well) and incubated for one hour at 4 °C. The cells were then washed twice with 300 µl 1 % SHEM (supplemented hormone epithelial medium containing 1% fetal calf serum) in order to remove unbound virions and then incubated in 1 ml 1 % SHEM for 44 h at 37 °C to allow infection. The wells were washed with phosphate-buffered saline (PBS, pH 7.4), fixed with ice-cold methanol (99 %), and incubated with a rabbit polyclonal anti-Ad37 serum (1:100 in PBS, 300 µl) at room temperature for one hour. The wells were washed with PBS and incubated

with swine polyclonal anti-rabbit FITC labeled antibody (DakoCytomation, Glostrup, Denmark, 1:100 in PBS, 300 μl) in room temperature for 1 hour. The wells were examined in an immunofluorescence microscope (Axioskop 2, Carl Zeiss, Germany; 10 x magnification). Data are presented as % of control, which is the value obtained in absence of inhibitor. Mean values and standard deviations (calculated with the Gauss approximation formula) are from duplicates of two independent experiments. The IC_{50} value for **ME0322** was calculated to 0.38 μM with Prism 5 (GraphPad Software, version 5.0d, trial).

Protein production and structure determination

Expression and purification were essentially carried out as described previously.[4] For co-crystallization Ad37 fiber knob trimers were concentrated to 18.2 mg/mL and then preincubated with a 1.3 fold excess of the trivalent sialic acid conjugate **ME0322**. Crystals of Ad37-**ME0322** complex were grown at 20 °C by the hanging drop method using a reservoir solution of 26 % PEG 8′000, 50 mM zinc acetate and 100 mM HEPES (pH 6.9). Crystals were then frozen in liquid nitrogen without cryoprotection and used to collect data on beamline MX 14-1 at BESSY II (Berlin, Germany). Diffraction data were recorded with a MX-225 charge-coupled device detector and processed with XDS-software.[5] The structure of Ad37 in complex with **ME0322** was solved first by molecular replacement using Phaser[6] in CCP4[7] and the native Ad37 knob trimer (pdb-code: 1uxe[8]) as the search model.

The terminal sialic acids were unambiguously placed in $F_{co\text{-}crystallized}$ - F_{native} difference Fourier maps, incorporated into the model, and refined with restraints from the Refmac5[9] monomer library. Structural refinement was carried out by alternating rounds of model building in Coot[10] and restrained refinement including the transition-libration-screw method (TLS) with Refmac5. For TLS refinement each protomer in the asymmetric unit (and the water chain) was attributed to one TLS group. The asymmetric unit contains two trimers of Ad37 fiber knob protein together with one molecule **ME0322** per Ad37 trimer. The crystallized Ad37 protein consists of amino acids 177-365, of which amino acids 182-365 are visible for chain A, E, F and G, amino acids 181-365 for chain B and amino acids 183-365 for chain C. Waters were located with ARP/wARP Solvent in CCP4.[7] Simulated Annealing was carried out with PHENIX.[11] The final model has excellent geometry. All figures were

prepared with PyMOL (DeLano Scientific). Statistics on data collection and refinement are given in Table S1 and the simulated annealing omit difference electron density map for one sialic acid of **ME0322** is given in Figure S1.

Surface Plasmon Resonance

The kinetic measurements were performed using a surface plasmon resonance BIAcore T100 instrument. Ad37 knob proteins were covalently coupled to a CM5 sensorchip using the amine coupling kit (GE Health care), to a concentration of 7-8 ng/mm^2. Binding of **ME0322** to the immobilized knob was performed in 20 mM Hepes, 0.15 M NaCl and 0.0005 % P20 pH 7.5. The concentrations of **ME0322** used were 35, 50 (twice), 75, 100, 150, and 200 μM (Figure S2). The association constant k_{ass}, dissociation constant k_{diss}, and affinity K_d were calculated using Biacore T100 evaluation software. k_{ass}: 20.39 $M^{-1}s^{-1}$; k_{diss}: 0.000288 s^{-1}; K_d (μM): 14.2 μM.

2. TABLE S1

Table S1. Data collection and refinement statistics.

	Ad37-ME0322
**Data collection**	
Space group	P $2_12_12_1$
Cell dimensions	
a, *b*, *c* (Å)	105.19, 108.65, 112.51
α, β, γ (°)	90.00, 90.00, 90.00
Resolution (Å)	35.00 – 2.40 (2.46 – 2.40) *
R_{merge}	11.4 (53.3)
$I / \sigma I$	16.7 (3.7)
Completeness (%)	99.5 (99.5)
Redundancy	7.3 (7.3)
Wilson factor (Å)	42.5
**Refinement**	
Resolution (Å)	2.40
No. of unique reflections	50848 (3691)
R_{work} / R_{free}	22.13 / 26.35
No. of non-H atoms	
Protein chain a / b / c / e / f / g	1450 / 1456 / 1435 / 1440 / 1443 / 1445
Sialic acid a / b / c / e / f / g	21 / 21 / 21 / 21 / 21 / 21
Zinc	7
Water	293
B-factors (Å)	
Protein chain a / b / c / e / f / g	41.7 / 34.1 / 46.5 / 39.6 / 37.6 / 37.5
Sialic acid a / b / c / e / f / g	32.4 / 35.8 / 37.8 / 29.2 / 29.1 / 35.3
Zinc	48.7
Water	29.8
R.m.s. deviations	
Bond lengths (Å)	0.006
Bond angles (°)	0.886

* Values in parentheses are for highest-resolution shell.

3. FIGURE S1 AND S2

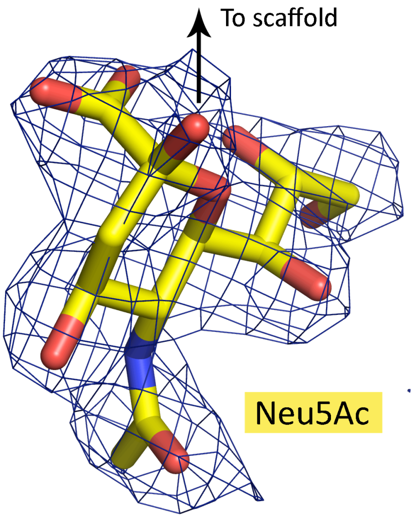

Figure S1. Simulated annealing omit difference electron density map for one sialic acid of **ME0322**, calculated at 2.4 Å resolution, contoured at 2.5 σ and displayed 4 Å around the sialic acid.

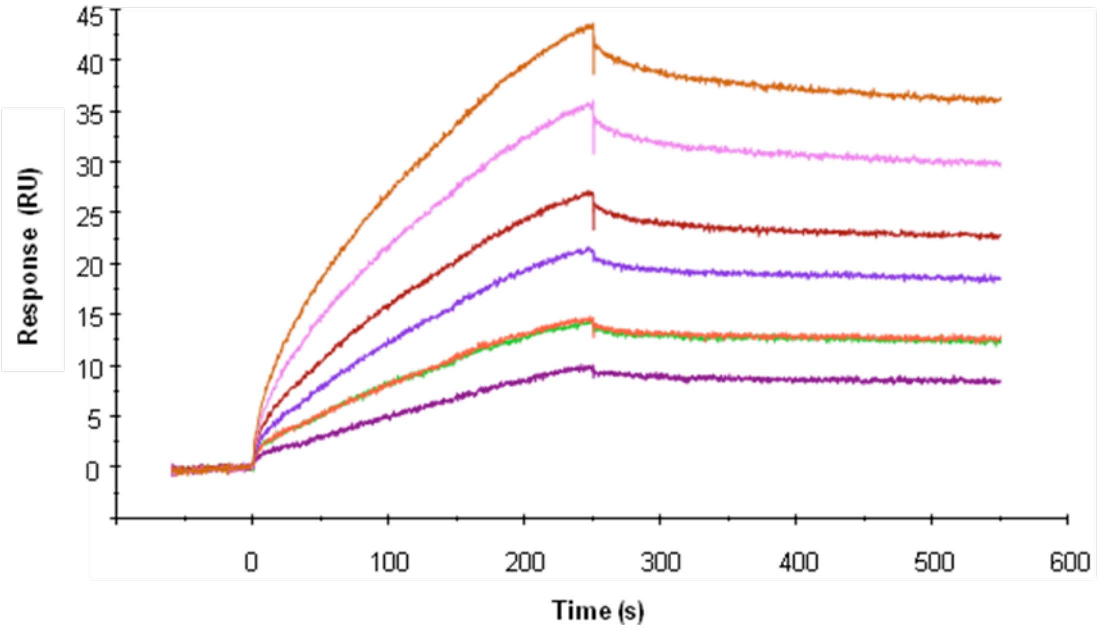

Figure S2. Surface plasmon resonance analysis of the interaction between the immobilized Ad37 fiber knob protein and **ME0322** at various concentrations. Dark purple: 35 µM; green: 50 µM; orange: 50 µM; blue: 75 µM; red: 100 µM; pink: 150 µM; brown: 200 µM. Knob binding over time is displayed as relative units (RU).

4. REFERENCES

[1] S. M. C. Johansson, N. Arnberg, M. Elofsson, G. Wadell, J. Kihlberg, *ChemBioChem* **2005**, *6*, 358-364.

[2] S. M. C. Johansson, E. C. Nilsson, M. Elofsson, N. Ahlskog, J. Kihlberg, N. Arnberg, *Antiviral Res.* **2007**, *73*, 92-100.

[3] S. Johansson, E. Nilsson, W. X. Qian, D. Guilligay, T. Crepin, S. Cusack, N. Arnberg, M. Elofsson, *J. Med. Chem.* **2009**, *52*, 3666-3678.

[4] E. C. Nilsson, R. J. Storm, J. Bauer, S. M. C. Johansson, A. Lookene, J. Ångstrom, M. Hedenström, T. L. Eriksson, L. Frängsmyr, S. Rinaldi, H. J. Willison, F. P. Domellöf, T. Stehle, N. Arnberg, *Nat Med* **2011**, *17*, 105-109.

[5] W. Kabsch, *J. Appl. Crystallogr.* **1993**, *26*, 795-800.

[6] A. J. McCoy, R. W. Grosse-Kunstleve, P. D. Adams, M. D. Winn, L. C. Storoni, R. J. Read, *J. Appl. Crystallogr.* **2007**, *40*, 658-674.

[7] *Acta Crystallogr., Sect. D: Biol. Crystallogr.* **1994**, *50*, 760-763.

[8] W. P. Burmeister, D. Guilligay, S. Cusack, G. Wadell, N. Arnberg, *J. Virol.* **2004**, *78*, 7727-7736.

[9] G. N. Murshudov, A. A. Vagin, E. J. Dodson, *Acta Crystallogr., Sect. D: Biol. Crystallogr.* **1997**, *53*, 240-255.

[10] P. Emsley, K. Cowtan, *Acta Crystallogr., Sect. D: Biol. Crystallogr.* **2004**, *60*, 2126-2132.

[11] P. D. Adams, P. V. Afonine, G. Bunkoczi, V. B. Chen, I. W. Davis, N. Echols, J. J. Headd, L.-W. Hung, G. J. Kapral, R. W. Grosse-Kunstleve, A. J. McCoy, N. W. Moriarty, R. Oeffner, R. J. Read, D. C. Richardson, J. S. Richardson, T. C. Terwilliger, P. H. Zwart, *Acta Crystallogr., Sect. D: Biol. Crystallogr.* **2010**, *66*, 213-221.
